# Prolonged storage-induced changes in haematology parameters referred for testing

**DOI:** 10.4102/ajlm.v4i1.208

**Published:** 2015-08-31

**Authors:** Elise Schapkaitz, Dashini Pillay

**Affiliations:** 1 Department of Molecular Medicine and Haematology, Charlotte Maxeke Johannesburg Academic Hospital, National Health Laboratory System Complex and University of Witwatersrand, South Africa; 2 Department of Haematology, National Health Laboratory Services and University of KwaZulu- Natal, South Africa

## Abstract

**Background:**

Referral of samples for the work-up of haematological disorders from remote laboratories can result in a delay in analysis.

**Objective:**

The stability of the full blood count (FBC), differential count (DIFF), reticulocyte and peripheral blood smear (PBS) morphology during extended storage was evaluated.

**Methods:**

Forty blood samples stored in ethylenediaminetetraacetic acid (EDTA) were analysed on an ADVIA® 120 haematology analyser. The samples (25% abnormal; 75% normal) were stored at room temperature (RT) and at 4 °C – 8 °C. Analysis of samples stored at RT was performed every 12 hours for two days. Analysis of samples stored at 4 °C – 8 °C was performed at 12 hours and subsequently every 24 hours for seven days.

**Results:**

FBC parameters (red cell count, haemoglobin) and DIFF parameters (percentages of basophils, lymphocytes and monocytes) were stable for at least 48 hours when stored at RT. Platelets were only stable for 12 hours and the white cell count was stable for 36 hours when stored at RT. Storing samples at 4 °C – 8 °C significantly increased the stability of most parameters, in particular, mean cell volume and percentage of reticulocytes. However, DIFF parameters were associated with lower stability at 4 °C – 8 °C. PBS morphology was compromised prior to 12 hours whether stored at RT or at 4 °C – 8 °C.

**Conclusion:**

This study provides evidence that blood samples stored in EDTA at 4 °C – 8 °C for seven days are suitable for testing on the ADVIA® 120 analyser for the FBC and percentage of reticulocyte parameters. However, storage at 4 °C – 8 °C is not a solution for samples referred for DIFF and PBS morphology review.

## Introduction

Pre-analytical variables, such as storage time and temperature, affect the measurement of laboratory parameters collected in ethylenediaminetetraacetic acid (EDTA).^1,2^ Laboratory staff need to be aware of the changes that occur during storage in their specific setting in order to decide whether to accept or reject samples that are too old to obtain reliable results. Accurate measurement of full blood count (FBC), differential count (DIFF) and reticulocyte parameters, as well as peripheral blood smear (PBS) morphology, are essential for the correct interpretation of haematology results.

It is recommended that traditional FBC parameters such as red cell count (RCC), white cell count (WCC), haemoglobin and platelet count be analysed 24 hours after sample collection when stored at room temperature (RT).^3,4,5^ However, parameters useful for diagnosis and monitoring of haematological disorders, such as mean cell volume (MCV), reticulocyte and PBS morphology, are unreliable after 12 hours.^5^ Osmotic swelling of red cells during storage at RT affects volume-dependant variables and results in misclassification of a microcytic anaemia as normocytic and, similarly, a normocytic anaemia as macrocytic.^6^ Reticulocytes mature into red cells after 24 hours in circulation. The Clinical and Laboratory Standards Institute (CLSI) recommends that samples stored at RT should be analysed for reticulocytes within six hours of collection.^7^ It is further recommended that PBS for morphologic analysis be prepared within four hours, prior to the onset of EDTA-induced changes in red and white cell morphology.^8,9,10^

With centralisation of laboratory services, it is not always feasible to meet these deadlines. Large academic laboratories are commonly faced with the scenario where a sample collected on Friday is not received in the laboratory for analysis until Monday morning. Recent studies indicate that longer storage durations are acceptable when samples are stored at 4 °C – 8 °C.^3,5,6,11,12^ However, information on stability beyond 72 hours is limited.^6^ Furthermore, these studies are small, specific to the haematology analyser, and the definition of stability used is not standardised.

The aim of this study was to evaluate the stability of the FBC, DIFF, reticulocyte and PBS morphology during extended storage at RT and at 4 °C – 8 °C in order to determine laboratory criteria for storage time and temperature for specimens referred for the work-up of haematological disorders from remote laboratories.

## Research method and design

### Ethical considerations

The study was approved by the Human Research Ethics Committee of the University of the Witwatersrand (M090688). All tests were done as part of routine diagnostic workups and no additional blood samples were taken from the participants for this study.

### Materials and setting

This study was conducted at the Main Haematology Laboratory of the Charlotte Maxeke Johannesburg Academic Hospital (CMJAH), National Health Laboratory Service Complex, Johannesburg, South Africa. Forty blood samples, representative of the patient population (25% abnormal and 75% normal specimens), that were left over after routine testing were selected from the haematology workload. Only samples collected in EDTA (Becton-Dickinson, Oxford, United Kingdom) vials with adequate volume (> 4 mL) received within two hours of collection were included. Samples with results that indicated partial aspiration were excluded from the final analysis.

### Design

#### Blood sampling and laboratory methods

Blood samples for evaluation of FBC, DIFF and reticulocyte parameters were collected in K_2_EDTA tubes (1.5–2.2 mg of dipotassium EDTA dihydrate per millilitre of blood). The parameters were analysed with the laboratory's ADVIA® 120 automated haematology analyser (Siemens Healthcare Diagnostics, Inc, Tarrytown, New York, United States). Cells were counted and sized by light scatter technology using white light for white cells and laser light for red cells and platelets. Haemoglobin was measured by the conventional cyanmethaemoglobin method. The six-part differential analysis was performed in two channels. Cells in the peroxidase channel were measured by peroxidase staining intensity and light scatter. Cells in the basophil/lobularity channel were measured by dual laser light scatter, nuclear density and lobulation index. Reticulocytes were stained with oxazine 750. The films were spread on the ADVIA® Autoslide slide maker and stained on the HEMA-TEK® 2000 slide stainer (Siemens Healthcare Diagnostics Inc, Tarrytown, New York, United States).

#### Laboratory testing

The samples were analysed within two hours of collection (time zero) at RT. Samples were aliquoted into two sets; one was stored at RT (18 °C – 24 °C) and and the other at 4 °C – 8 °C. Analyses of samples stored at RT were performed after 12, 24, 36 and 48 hours of storage. Analyses of samples stored at at 4 °C – 8 °C were performed after 12, 24, 36, 48, 72, 96, 120, 144 and 168 hours of storage.

A manual DIFF was performed on PBS of five samples stored at RT and at 4 °C – 8 °C. Reviews were performed at 12, 24, 36 and 48 hours. The PBS was first examined for the presence of EDTA-induced changes, including red cell spherocytes, echinocytes, sphero-echinocytes, increased rouleaux formation, degeneration of neutrophils and lobulation of lymphocyte nuclei,^10^ because these changes preclude an accurate manual DIFF.

#### Analyses

Data were captured from the analyser printouts on Excel™ spreadsheets (Microsoft Office Excel™ 2007, Redmond, Washington, United States) and analysed using Statistica 9.1 software (StatSoft, Tulsa, Oklahoma, United States). The mean percentage difference from the value at time zero was calculated and tabulated.^13^ Stability of a parameter was defined in relation to the precision of the ADVIA® 120 analytical method. Acceptable limits were defined in accordance with the Royal College of Pathologists of Australasia external quality assurance annual review for 2013.^14^ The coefficients of variation (% CV) for the parameters were as follows: WCC 3.4%, RCC 1.8%, haemoglobin 1.8%, haematocrit 2.4%, platelet count 2.4% and percentages of neutrophils 0.9%, lymphocytes 4.9%, monocytes 5.6%, eosinophils 16.7%, basophils 55% and reticulocytes 8.1%. A parameter was considered stable, when its difference was smaller than 1% CV for the assessed method.^5^

## Results

### Storage at room temperature

**FIGURE 1:**
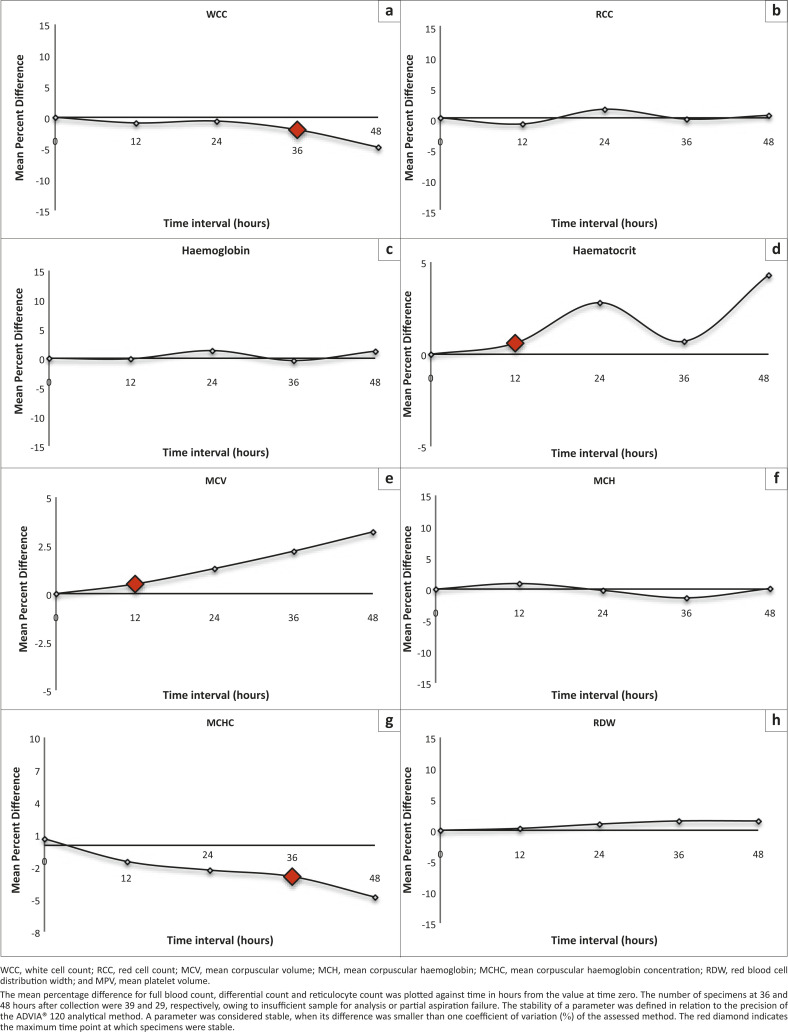

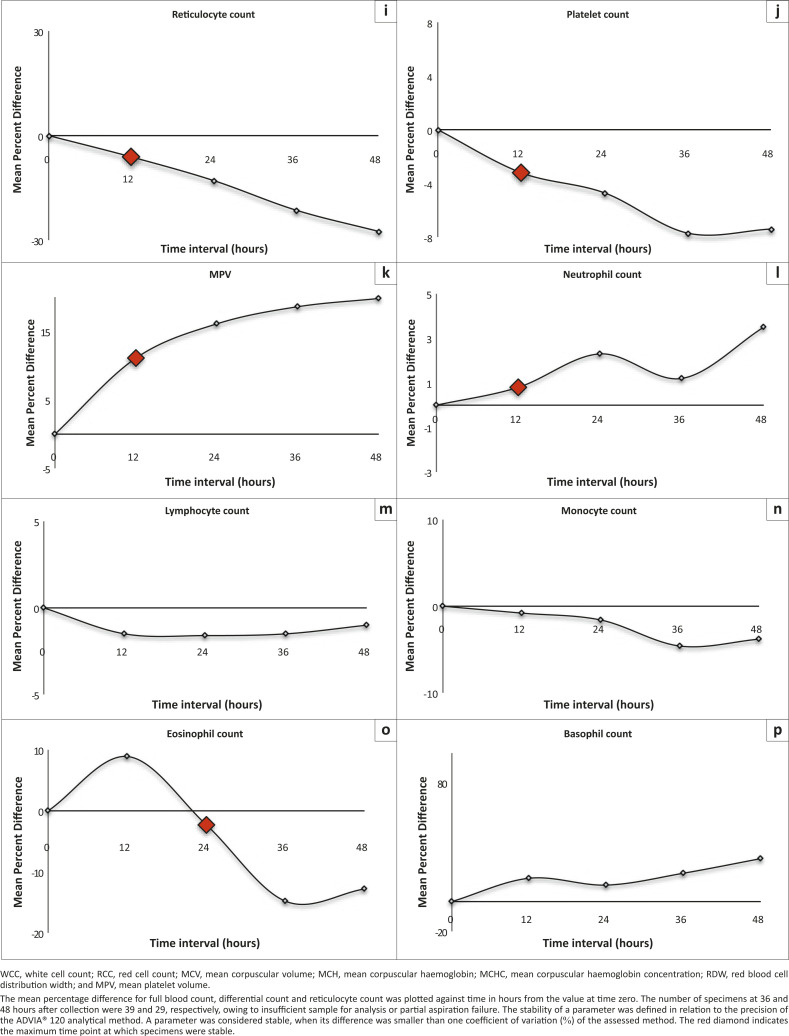
Stability analysis (mean percent difference) at room temperature (18 °C – 24 °C)

The WCC was stable until 36 hours after collection and showed a significant decrease at 48 hours after collection (Figure 1). A significant increase in the percentages of neutrophils and eosinophils was observed at 24 hours and 36 hours, respectively. Red cell parameters including RCC, haemoglobin, mean cell haemoglobin (MCH) and red cell distribution width (RDW) were stable for at least 48 hours after collection when stored at RT and were not significantly affected by storage temperature. In contrast, other RCC measurements, including haematocrit, MCV, mean cell haemoglobin content (MCHC) and the percentage of reticulocytes, were not stable after storage at RT for 48 hours after collection. After RT storage for 24 hours, a significant increase in MCV, as well as a decrease in the percentage of reticulocytes, was observed. Analysis of platelet stability showed platelets were stable for 12 hours and significantly decreased at 24 hours after collection. At RT storage, the stability of the mean platelet volume (MPV) was less than 12 hours as a result of artificial platelet swelling. At RT storage, the stability of the manual DIFF was also less than 12 hours. The percentages of neutrophils and monocytes showed significant increases, whereas percentages of lymphocytes and eosinophils showed significant decreases at 12 hours after collection. The slides examined contained too few basophils to obtain reliable results for basophil stability. EDTA-induced changes were noted at 24 hours after collection, which precluded a manual DIFF.

### Storage at 4 °C – 8 °C

**FIGURE 2:**
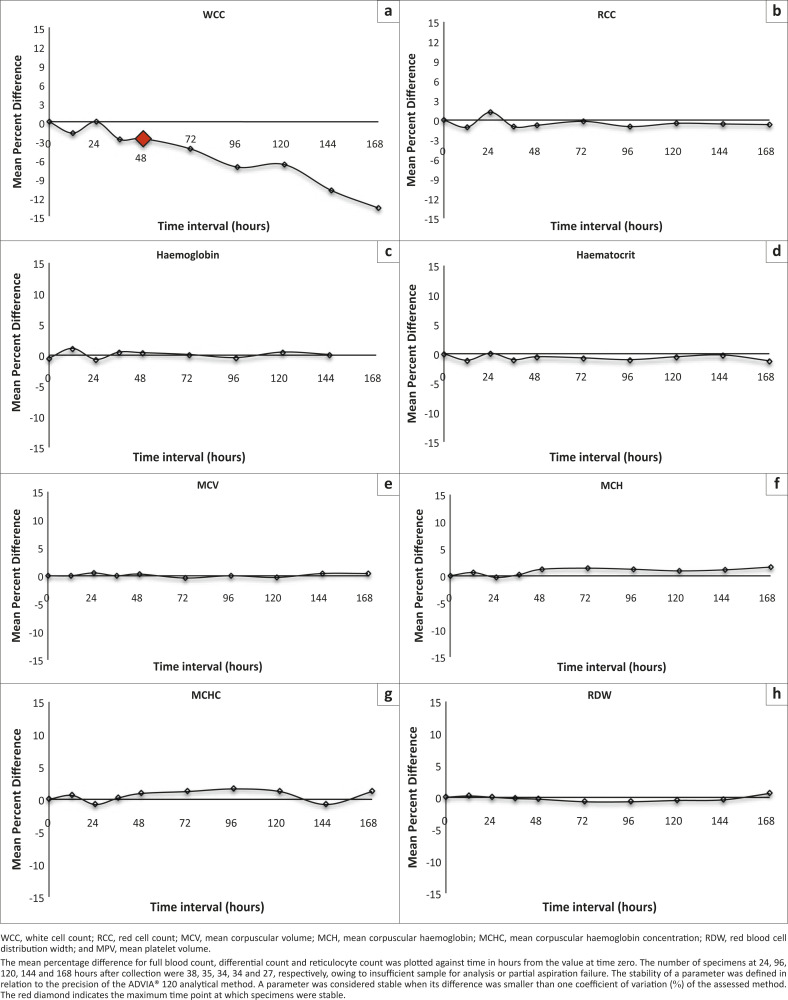

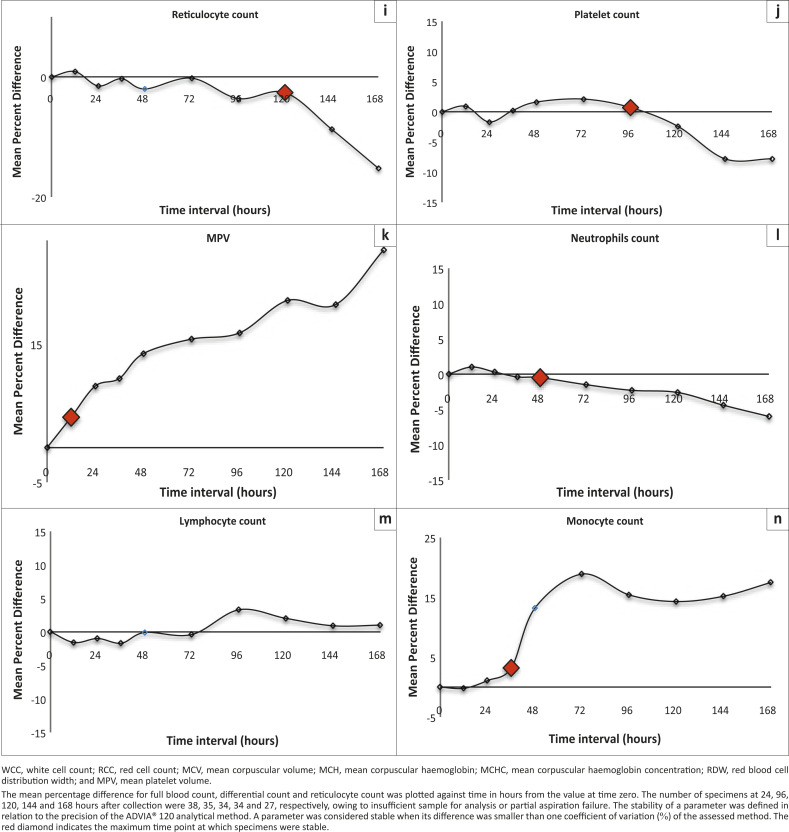

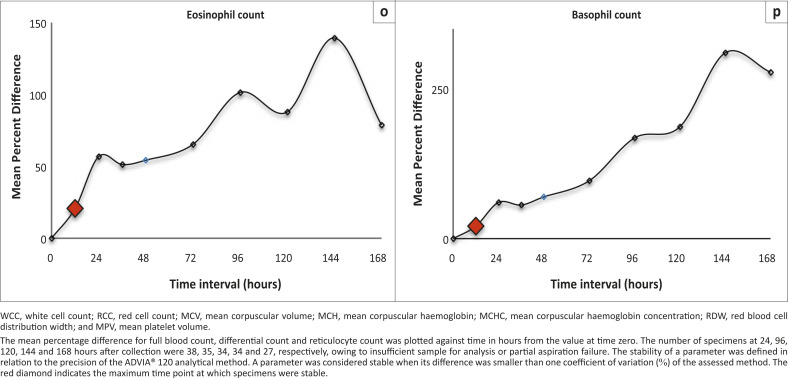
Stability analysis (mean percent difference) at 4 °C – 8 °C.

The WCC was stable at 4 °C – 8 °C until 48 hours after collection (Figure 2). A significant decrease in the percentage of neutrophils was observed at 72 hours after collection. The percentages of eosinophils, basophils and monocytes were not stable when stored at 4 °C – 8 °C and showed significant increases at 12, 24 and 48 hours, respectively. Compared with RT storage, we observed improved stability of RCC parameters when stored at 4 °C – 8 °C. Haematocrit, MCV and MCHC were stable until 168 hours when stored at 4 °C – 8 °C. The percentage of reticulocytes was stable until 120 hours after collection and showed significant decreases thereafter. Platelets were stable until 96 hours after collection when stored at 4 °C – 8 °C. MPV showed a significant increase at 24 hours after collection. As a result of the presence of EDTA-induced changes prior to 12 hours after collection, a manual DIFF could not be performed.

## Discussion

Routine tests such as the FBC, DIFF, reticulocyte and PBS morphology are commonly referred to the CMJAH haematology laboratory as part of the diagnostic work-up for haematological disorders. In large academic laboratories, where aged samples make up a significant proportion of the workload, the storage time and temperature of samples must be taken into consideration. The findings of this study performed on EDTA samples add to the evidence that stability varies according to storage time and temperature.

According to the findings of this study, FBC parameters, namely RCC, haemoglobin, MCH and RDW, and DIFF parameters, namely percentages of basophils, lymphocytes and monocytes, were least affected by storage temperature and time and can be analysed until 48 hours after sample collection when stored at RT.

It is recommended that traditional FBC parameters be analysed 24 hours after sample collection when stored at RT.^3,4,5^ However, in this study, platelets were only stable until 12 hours after collection when stored at RT. The stability of the WCC was also found to be shorter than other studies, which have recommended analysis up to 48 hours after collection when stored at RT.^5,11,15^ In this study, the WCC was stable only until 36 hours after collection when stored at RT.

In this study, the MCV was stable until 12 hours after collection. Imeri et al. found that MCV increased significantly after 4–10 hours, regardless of the haematology analyser;^5^ whereas other studies have indicated a longer stability of up to 24 hours for MCV at RT.^3,11,15^ These discordant results may be attributed to the different statistical methods used in these studies for the evaluation of stability, which limits accurate comparison.

In this study, the percentage of reticulocytes was stable at RT until 12 hours after collection, which concurs with current recommendations.^3,5^ However, the stability of reticulocyte parameters has been found to be longer at RT on other haematology analysers, such as the Coulter® LH 750, Sysmex XE-2100™ and Cell-DYN Sapphire™.^5,11^ According to Wiegand et al., the oxazine 750 on the ADVIA® haematology analysers may not be sufficient to detect more mature reticulocytes.^16^

Imeri et al. conducted a three-way comparison study of the Coulter® LH750, Sysmex XE-2100™ and ADVIA® haematology analysers that further illustrated how the stability of many haematology parameters depends on the analytical method used.^5^ Thus, the findings of this study are specific to ADVIA® haematology analysers, which currently represent 60% of haematology analysers in South Africa. Therefore, the findings of this study have widespread local implications. However, CLSI do recommend that ‘laboratories should assess FBC stability in their specific settings’.^17^

Storage of samples at 4 °C – 8 °C for seven days increased the stability of most parameters. FBC parameters, namely WCC, platelet count, haematocrit, MCV and MCHC, as well as DIFF parameters, namely percentages of neutrophils and reticulocytes, were more stable when stored at 4 °C – 8 °C. However, some DIFF parameters, namely percentages of eosinophils, basophils and monocytes, had lower stability.

Changes were present on PBS morphology prior to 12 hours after collection when stored at either RT or at 4 °C – 8 °C. This precluded assessment of dysplastic morphological features. It is currently recommended that PBS be prepared within a few hours for assessment of haematological disorders, in particular for the presence of dysplastic features.^9,10,18^

## Limitations of the study

A limitation of this study is that PBS morphology was not evaluated prior to 12 hours after collection (i.e., at four and six hours). As such, a manual differential could not be performed. Furthermore, this study was performed under optimal conditions on inpatient samples that were received in the laboratory within two hours of collection. Samples referred for testing are often subject to variation in temperature during collection and transport.

## Conclusion

In conclusion, this study provides evidence regarding the viability of blood samples collected in EDTA vials and stored at RT and at 4 °C – 8 °C. Samples that have been stored at 4 °C – 8 °C for seven days are suitable for testing on the ADVIA® 120 analyser for FBC and reticulocyte parameters. However, this is not a solution for samples referred for DIFF or PBS morphology review.
